# Formulation and Evaluation of Transdermal Gel Containing Tacrolimus-Loaded Spanlastics: *In Vitro*, *Ex Vivo* and *In Vivo* Studies

**DOI:** 10.3390/polym14081528

**Published:** 2022-04-09

**Authors:** Randa Mohammed Zaki, Mohamed A. Ibrahim, Doaa H. Alshora, Amal El Sayeh Abou El Ela

**Affiliations:** 1 Department of Pharmaceutics, Faculty of Pharmacy, Prince Sattam Bin Abdulaziz University, Al-Kharj 11942, Saudi Arabia; randazaki439@yahoo.com; 2 Department of Pharmaceutics and Industrial Pharmacy, Faculty of Pharmacy, Beni-Suef University, Beni-Suef 62514, Egypt; 3 Department of Pharmaceutics, College of Pharmacy, King Saud University, Riyadh 11451, Saudi Arabia; mhamoudah@ksu.edu.sa (M.A.I.); aelsaih@ksu.edu.sa (A.E.S.A.E.E.); 4 Department of Pharmaceutics and Industrial Pharmacy, College of Pharmacy, Al-Azhar University, Assiut 71524, Egypt; 5 Department of Pharmaceutics, College of Pharmacy, Assiut University, Assiut 71526, Egypt

**Keywords:** tacrolimus, spanlastics, transdermal gel, drug permeation, penetration enhancers

## Abstract

Our goal was to prepare Span 60-based elastic nanovesicles (spanlastics (SPLs)) of tacrolimus (TCR) using the adapted ethanol injection method, characterize them, and evaluate their ability to improve the transdermal permeation of the active substance. The impact of two different concentrations of penetration enhancers, namely, propylene glycol and oleic acid, on the entrapment efficiency, vesicle size, and zeta potential was assessed. Moreover, in vitro release through a semipermeable membrane and ex vivo penetration through hairless rat skin were performed. Morphological examination and pharmacokinetics were performed for one selected formulation (F3OA1). TCR-loaded SPLs were effectively formulated with two different concentrations of permeation enhancers, and the effect of these enhancers on their physicochemical properties differed in accordance with the concentration and kind of enhancer used. The results of in vitro release displayed a considerable (*p* < 0.05) enhancement compared to the suspension of the pure drug, and there was a correlation between the in vitro and ex vivo results. The selected TCR-loaded nanovesicles incorporated into a gel base showed appreciable advantages over the oral drug suspension and the TCR-loaded gel. Additionally, the pharmacokinetic parameters were significantly (*p* < 0.05) improved based on our findings. Moreover, the AUC_0–7_ ng·h/mL form F3 OA1 was 3.36-fold higher than that after the administration of the TCR oral suspension.

## 1. Introduction

Tacrolimus (TCR) is an immunosuppressant, which was approved by the FDA in 1994 for oral administration, to prevent the rejection of liver and kidney transplants [1]. TCR acts as a calcineurin inhibitor by binding to specific receptors on the T cell that inhibit its activation and the production of various cytokines [2]. TCR is characterized by poor solubility, which is why it belongs to the Class II biopharmaceutical classification system (BCS). This poor solubility contributes to some extent to lowering the bioavailability. After oral administration, TCR is highly subjected to first-pass metabolism. Therefore, the bioavailability of the drug does not exceed 14% [3]. TCR has a large molecular weight (822.95 g/mol) and high lipophilicity (partition coefficient, log P = 3.96 ± 0.83); these properties limit its transport through skin tissue [4]. Several studies have been conducted to improve the permeation of the drug through the stratum corneum. Yamanaka et al. [5] showed that the AUC increased by 4.6-fold after the topical administration of TCR oil/water emulsion-based cream with isopropyl myristate [5]. TCR-loaded hydrogel composite nanocarriers have also shown a significant 2-fold improvement in the topical delivery of TCR through the skin compared to commercial products [6].

Spanlastics (SPLs) are novel drug delivery systems that combine vesicular and nanoparticulate properties. The term “Spanlastic” comes from span + elastic, where the drug (core) is enclosed in the form of a bilayer. SPLs are made from a surfactant, mainly span, and edge activators such as Tween 80 and ethanol, which have the dual effect of improving the entrapment of the drug in the vesicle and increasing the stability by steric stabilization [7]. The choice of surfactant type can affect the stability of vesicles. Most studies have found that Span 60 has a significant effect on producing more stable vesicles than Spans 40 and 80 [8,9,10,11]. Polysorbate 80 (Tween^®^ 80) is mainly used as an edge activator that increases the flexibility of vesicles, which leads to better drug penetration by increasing the opening magnitude of the biological membrane [12,13]. SPLs offer several advantages over the conventional vesicular system as they are more stable and biodegradable. In addition, the fabricated systems are elastic and deformable and, therefore, can be modulated as desired [7]. SPL-based drug delivery systems have been used to enhance the permeation and delivery of various drugs, such as epigallocatechin gallate from green tea [14], fenoprofen calcium [15], and ascorbic acid [16].

The transdermal drug delivery may offer several advantages over oral delivery. It eliminates the degradation of the drug by first-pass metabolism and delivers the drug over a longer period of time with less fluctuation [17]. The permeation and penetration of the drug through the stratum corneum may perform as a rate-restricting phase in many transdermal delivery systems. Therefore, penetration enhancers are added to improve drug penetration [18]. Fatty acids are well-known penetration enhancers. Their effect depends on their structure, degree of saturation, and the extent of the alkyl chain [19]. According to the FDA, fatty acids are generally recognized as safe (GRAS), and their use is approved. The addition of permeation enhancers to SPLs may have a synergistic effect in improving drug penetration into the skin and eyes. Although there are insufficient data to explain how far this synergistic effect extends, some articles have shown that it has a positive effect on the penetration of active substances. Haloperidol has been formulated as a spanlastic with Labrasol, Transcutol P, or tetraglycol as penetration enhancers. The results showed that Labrasol increased the diffusion of the drug and the prepared spanlastics had optimal criteria [20].

Gel formulations are semisolid preparations in which a liquid phase is constrained within a three-dimensional polymeric matrix. Gels are becoming more common because of their ease of application, sustaining drug release, and evasion of the various conditions of absorption, such as gastric pH, first-pass metabolism, and gastric enzymes. Moreover, the patient can terminate the medications at any time. Incorporation of nanospanlastics into hydrogel provides easy application and sustained drug delivery, consequently, improving patient compliance. Additionally, as a sustained release system, it prolongs the duration of the drug concentration in the therapeutic range, while it also has a reduced dose of the drug [21,22].

In the present study, TCR was loaded into SPLs as an alternative delivery system to improve its skin permeation. Different ratios between Span 60 and Tween 80 (edge activator) were investigated. The loading of the active substance was performed in the presence of two different permeation enhancers (propylene glycol and oleic acid) by using a modified ethanol injection technique. The effect of the two different permeation enhancers’ concentrations was studied. The prepared TCR-loaded SPLs were characterized in terms of particle size, zeta potential, entrapment efficiency (EE%), in vitro release, and ex vivo permeation through hairless rat skin. One selected formulation was further incorporated into a gel base and used for an in vivo study in rats.

## 2. Materials and Methods

### 2.1. Materials

TCR was kindly supplied by Adwia Pharmaceuticals Co. (Cairo, Egypt). Sorbitan monoesters (Span 60) were obtained from Loba Chemie (Mumbai, India). Polysorbates (Tween^®^ 80) were obtained from BDH (Kingston, England). Hydroxypropyl methylcellulose (HPMC) were obtained from Colorcon, UK. Propylene glycol (PG) was purchased from Winlab (Germini-house, England). Oleic acid (OA) was obtained from Lobachemi (Mumbai, India). Chloroform, ethanol, and acetonitrile (HPLC grade) were purchased from Fisher Scientific Co. (Hampton, NH, USA). All other ingredients were of pure analytical grade.

### 2.2. Methods

#### 2.2.1. Preparation of TCR-Loaded SPLs

The adapted ethanol injection technique was applied to the formulation of SPLs [23] using Span 60–Tween 80 in ratios (mg) of 85:15, 80:20, 75:25, 70:30, and 50:50. All SPL formulations contained 5 mg of TCR. Span 60 and TCR were dissolved in 10 mL of the organic phase consisting of chloroform–ethanol (2:1, *v*/*v*). Propylene glycol and oleic acid were used as permeation enhancers at two different concentrations: 0.5% and 1% (*w*/*v*). The organic phase was gradually added to 10 mL of the heated (75 °C) aqueous phase (Ultrapure water containing the edge activator and the penetration enhancer), which was continuously stirred for 60 min with a magnetic stirrer at 800 rpm to evaporate the organic phase and obtain the final volume of 10 mL. In this way, a homogeneous, milky-white suspension of SPLs free of visible particles was produced. The resulting suspension was cooled, and once a stable SPLs suspension was produced, it was subjected to probe-ultrasonic exposure (Badnelin, Germany) at 25 °C for 5 min to obtain a nanosized vesicles. The composition of the different SPL formulations is shown in Table 1.

#### 2.2.2. In Vitro Characterization of TCR-Loaded SPLs

##### Size of the TCR-Loaded SPLs

The size of the SPLs of the obtained formulations was determined by the dynamic light scattering technique (DLS), using Malvern Zetasizer (Nano ZS, Malvern, UK) at a room temperature of 25 °C, after diluting the formulation (0.1 mL made up to 1 mL using Millipore water). Three size determinations for every formulation were conducted, and the mean ± SD was determined. The polydispersibility index (PDI) was determined with each size measurement.

##### Zeta Potential

The Zetasizer ZS (Malvern Instruments, UK) was used for the estimation of the charges on the vesicular exterior surface of the SPLs at 25 °C for 120 s utilizing a combination of laser Doppler velocimetry and phase analysis light distribution to evaluate particle electrophoretic mobility. Then, after diluting the formulation (100 μL dispersed into 1 mL Millipore water) at 25 °C, the measurements were obtained. The mean zeta potential of the three experiments of each formulation was shown.

##### Entrapment Efficiency Percent (EE)

TCR-loaded SPLs were separated from the liberated drug during preparation using the ultracentrifugation method using a cooling centrifuge (Optima^TM^ Max-E, Ultracentrifuge, Beckman Coulter, Pasadena, CA) at 17,000 rpm and a temperature of 4 °C for 60 min. Then, the supernatant was collected and filtered through a 0.45 μm syringe filter (Millipore Milex-HN, Merck, Germany). The filtered solution was diluted 10 times with the mobile phase, and TCR was computed using HPLC analytical procedures. Drug EE was calculated using three measures, and the mean ± standard deviation (SD) corresponding to the subsequent equation was estimated [24]:

$$\%\mathrm{EE}=\frac{\mathrm{Total}\;\mathrm{amount}\;\mathrm{of}\;\mathrm{drug}\;\cdot \;\mathrm{Free}\;\mathrm{drug}\;\mathrm{in}\;\mathrm{the}\;\mathrm{supernatant}\;}{\mathrm{Total}\;\mathrm{amount}\;\mathrm{of}\;\mathrm{drug}\;}\times 100$$

The samples were analyzed using a sensitive HPLC method. The HPLC was performed with WatersTM 600 controller, (Milford, MA, USA) equipped with WatersTM 1252 a Binary pump and dual-absorbance detector (WatersTM 2487). The analysis was achieved at 50 °C using C18 column (100 Å, 150 × 4.6 mm, 5µm) (Youngjin Biochrom Co., Ltd., Seongnam, Korea) and mobile phase containing a mixture of acetonitrile and distilled water solution (70:30 *v*/*v*), at flow rate of 1.1 mL/min. The mobile phase was filtered with 0.22 Nylon filter (Sartorius, Gottingen, Germany) and degassed before being used. The detector was set at 210 nm and the injection volume was 20 µl. The concentration range for the calibration curve was from 20 to 100 ng/mL, and the recovery ranged from 97.13% to 102.04%. The linearity was between 20 and 100 ng/mL (R^2^ = 0.998; *n* = 6).

##### Transmission Electron Microscopy (TEM)

The morphology and structure of the selected TCR–SPLs (F3 OA 1) were studied using TEM (TEM 1230; JEOL, Tokyo, Japan). A drop of the TCR–SPL suspension was employed to immerse the copper grid for 1 min. Consequently, the particles on the grid were stained with 2% (*w*/*v*) phosphotungstic acid solution for 10 s and cleansed two times with distilled water for 1 s. The grids were dehydrated in a dust-free atmosphere for 30 min and then studied by TEM accurately at an acceleration voltage of 100 Kv.

##### Differential Scanning Calorimetry (DSC)

The thermal characteristics of the TCR pure substance and chosen lyophilized TCR-loaded SPLs (F3 OA 1) were investigated using a differential scanning calorimeter (Shimadzu, Kyoto, Japan, DSC–60). The TCR–SPL formula was lyophilized using a freeze-dryer (Martin Christ, Osterode, Germany), which removed all water to leave a dry powder. The preparations were precisely weighed (2 mg), packed into an aluminum pan, and inspected at a heating rate of 10 °C/min in a temperature range of 20 °C to 200 °C with a nitrogen supply of 50.0 mL/min. The created thermograms were observed and assessed for any disappearance or appearance of new peaks.

##### Formulation of TCR-Loaded SPL Gel Bases

Based on the characterization results from all the prepared SPLs, one formulation (F3 OA 1) was further incorporated into a gel base. Hydroxypropyl methylcellulose (HPMC, K4M) was chosen as gelling polymer at a concentration of 2.5%. The gel was prepared by dispersing the required quantity of HPMC (0.25 g) in a sufficient quantity of distilled water (10 mL) under constant stirring at 1000 rpm until a homogenous system was obtained. The TCR–SPLs (F3 OA 1) was prepared as previously described, and the unentrapped drug was removed by ultracentrifugation. The residue (TCR-loaded SPLs) was dispersed in the gel base so that the final formulation contained 5 mg of TCR per 1 g of gel base.

Characterization of TCR-Loaded SPLs Gel Base

The formulated gel was inspected visually for different physical parameters such as clarity, homogeneity, and phase separation. The pH of the TCR-loaded SPLs gel formulation was measured by means of a digital pH meter (PHASAR-1, Beckman, USA). One gram of the gel was diluted and dispersed well in 10 mL of the distilled water, then pH was measured in triplicate. The spreadability of the TCR-loaded SPLs gel formulation was assessed by placing 0.5 g of the gel in the center of a watch glass (5 cm in diameter), and then another watch glass was gently located over the first one and left for 5 min to make sure that there was no expectation of further spreading. The diameter of the spread circle was assessed and considered as comparative values for spreadability.

##### In Vitro Release Studies

In vitro release experiments of the SPL formulations were performed by applying Franz diffusion cells (Logan Instruments, Somerset, NJ, USA) at 37 ± 0.5 °C and 100 rpm. The release was performed for F3 containing Span 60–Tween; 75:25; F3 containing 0.5% propylene glycol (F3 PG 0.5); F3 containing 1% propylene glycol (F3 PG 1); F3 with 0.5% oleic acid (F3 OA 0.5) and F3 prepared with 1% oleic acid (F3 OA 1) (Table 1)). Moreover, the in vitro release of pure drug suspension in water equivalent to 5 mg TCR (1 mL) was used as a control for the TCR-loaded SPL formulations. A semipermeable membrane from Sigma Chem. Co. (MO, USA) with a molecular cut-off of 14,000 Da was employed for the release experiments. Ethanol and pH 7.4 phosphate-buffered solutions (PBS) were mixed in a ratio of 3:7 (*v*/*v*) to maintain the sink condition and manipulated as the acceptor phase. A quantity of the lyophilized TCR–SPLs (equivalent to 5 mg TCR) was placed on the donor chamber and protected with parafilm to avoid dehydration. Samples (1 mL) were withdrawn at intervals of 0.5, 1, 2, 3, 4, 5, 6, and 7 h, and the volume of each removed sample was substituted with an equal volume of fresh buffer. The concentration of TCR in the samples from the acceptor medium was evaluated using HPLC, as illustrated earlier.

The in vitro release of TCR from the selected gel base containing the SPLs formulation (F3 OA 1) was also undertaken with a Franz diffusion cell by placing a particular quantity (1 gm) of the selected gel formulation containing TCR equivalent to 5 mg on the presenter compartment, as previously explained. All experiments were performed in triplicate. The percentage of the drug liberated was assessed and drawn with regard to time. The data were fitted into various kinetic models: zero-order, first-order, second-order, and Higuchi models. The correlation coefficient (R^2^) was determined for each case.

#### 2.2.3. Ex Vivo Permeation Study

The ex vivo permeation of TCR from SPL dispersions through hairless rat skin was carried out by using Franz diffusion under occlusive conditions, with an effective permeation area of 3.14 cm^2^ and receptor volume of 12 mL [24]. The animal use was approved by the Research Ethics Committee, Department of Pharmacology and Toxicology, Faculty of Pharmacy, Prince Sattam Bin Abdulaziz University, Al-Kharj, Saudi Arabia. (Approval No: BERC-004-03-21). The rats (150–220 g) were anesthetized and sacrificed, and the hair on the abdomen was carefully shaved off. The abdominal skin was excised, the subcutaneous tissues were removed surgically and rinsed with standard saline, and then the skin samples were stored at −20 °C until use. The skin was fixed between the donor and acceptor compartments with the stratum corneum facing the donor compartment. The ex vivo permeation experiment was performed as mentioned above for the in vitro release of TCR-loaded SPLs (equivalent to 5 mg TCR). All samples were filtered through a 0.45 μm Millipore membrane filter, and the concentration of TCR in the samples from the donor chamber was studied using HPLC, as previously described. Moreover, the ex vivo permeated amount of TCR from the drug suspension containing 5 mg TCR in 1 mL water was determined. All investigations were performed in triplicate, and the percentage of drug permeated was calculated.

Concerning the ex vivo permeation of the gel base containing the selected SPLs formulation (F3 OA 1), the procedure was also performed with a Franz diffusion cell under occlusive circumstances as prescribed above. All experiments were performed in triplicate, and the percentage of drug permeated was calculated. The permeation parameter of TCR, the skin permeation rate of drug flux (J_ss_, μg cm^−2^ h^−1^), was estimated from the slope of the plot of the cumulative quantity of drug penetrated against time at a steady state divided by the area of diffusion. The permeability coefficient (Kp, cm/h) was estimated from the ex vivo permeation data obtained from the rat skin and was calculated with the equation Kp = J_ss_/C_o_, where C_o_ is the original concentration of the drug in the donor compartment. Moreover, the partition coefficient of the drug (K) between the rat skin and the delivery system was also determined. [25].

#### 2.2.4. Pharmacokinetics of TCR from SPL Gel Base

The in vivo study was carried out in rats to examine the capability of the transdermal SPLs gels in improving TCR pharmacokinetic parameters. Male albino rats weighing approximately 300 g were utilized for the study. The rats fasted for 12 h before the study with free access to water and were divided into three groups. Group I, the positive control, received 0.6 mL oral TCR suspension in water (5 mg/mL) by intraesophageal intubation at a dose of 10 mg/kg [26]. Group II and III were subjected to topical untreated TCR and the selected TCR-loaded SPL gel, respectively. After shaving the back hair in one square spot, 0.6 g of TCR-loaded gel and TCR-loaded SPL gel were applied topically at a dose of 10 mg/kg. At specified time intervals (0.5, 1, 2, 4, 6, 8, 12, and 24 h), blood samples of approximately 0.5 mL were withdrawn from the retro-orbital puncture of each rat and kept in heparinized tubes until further investigation.

##### Sample Preparation for Pharmacokinetic Analysis

TCR is highly bound to red blood cells (RBCs) and plasma protein. Therefore, whole blood samples are preferred as test samples for TCR content. In this context, to estimate the TCR concentration, RBCs in the blood samples must be lysed by a hemolytic agent, and plasma proteins must be denatured [27]. For TCR extraction from blood samples, approximately 250 µL of blood samples was mixed well with 125 µL zinc sulfate solution (0.2 M) as a hemolytic agent to lyse the cells followed by vortexing for 15 sec with 250 µL of acetonitrile solution of ascomycin (internal standard) containing 100 ng/mL. An amount of 3 mL of ethyl acetate was added to the samples and then vortexed for 1 min. The resultant solutions were then centrifuged for 10 min at 6000 rpm. After that, the organic layers were separated and evaporated under nitrogen at 45 °C. The residues were dissolved in 100 mL of the mobile phase for injection. The TCR concentration of the blood samples was determined utilizing the previously mentioned HPLC method.

##### Pharmacokinetic Parameters (PK)

Pharmacokinetic parameters after the oral and transdermal administrations were assessed by WinNonlin, version 1.5 (Scientific Consulting, Inc., Cary, NCT). Parameters such as the maximum drug concentration C_max_ (ng/mL); the time required to achieve such a concentration T_max_ (h); the area under the curve, AUC_0–24_ (ng h/mL); AUC from 0 to infinity, AUC_0–∞_ (ng h/mL); half-life t_½_; and mean residence time MRT_0–∞_ (h) were determined.

#### 2.2.5. Statistical Data Analysis

Data analysis was carried out with the software package Microsoft Excel, Version 2010, and origin software, version 8. Results are presented as the mean ± SD (*n* = 5). A difference below the probability level of 0.05 was considered to be statistically significant.

## 3. Results and Discussion

### 3.1. Characterization of TCR-Loaded SPLs

The prepared TCR–SPL formulations were effectively prepared using the ethanol injection method. The saturated lipophilic alkyl chain in Span 60, a lipophilic and nonionic surfactant, may facilitate the formation of mono- and/or multilamellar vesicles. Tween 80 and permeation enhancers (propylene glycol and oleic acid) promote vesicle flexibility in some formulations, creating systems with disrupted packing properties that are able to force themselves into the openings of the skin [20]. In addition, the permeation enhancers have the ability to temporarily enlarge the skin openings, allowing for the better penetration of the SPLs [28].

Table 2 shows the EE percent, mean vesicle size (nm), PDI, and zeta potential distribution (mV) of the different SPL vesicles. The results revealed that an increase in the Tween ratio in the SPL formulations tested led to an increase in vesicle size. The measured vesicle size of formulation F1 (Span 85:15 Tween) was 38.1 ± 7.01 nm, whereas it was 407.3 ± 8.4 nm for formulation F5 (Span 50:50 Tween) (Table 2). This phenomenon could be due to the affinity of Tween as a hydrophilic surfactant towards the aqueous medium during the preparation of the SPLs, resulting in an increase in SPLs size. Fahmy et al. [20] showed that increasing the amount of Tween 80 in haloperidol SPLs resulted in a significant increase in the size of the vesicles. The size of the vesicles of the formulated SPLs varied from 38.1 ± 7.01 nm to 407.3 ± 8.4 nm. This range of vesicle size is suitable for transdermal drug delivery [20,29]. The formulated SPLs had a PDI of less than 0.4, indicating a moderately homogeneous size distribution [14]. Moreover, the zeta potential values ranged from −16 ± 1.3 mV to −24.5 ± 0.7 mV. The largest absolute value was obtained by (F3 OA 1), which gave stable TCR-loaded SPLs due to electrical repulsion.

TCR was successfully encapsulated in all the SPLs prepared with different ratios of Tween 80 and Span 60. These highest values of entrapment efficiency mean that the drug is targeted to the skin surface and has fewer systemic side effects [30]. The results showed that the EE percent of SPLs was independent of the Span–Tween ratio. The F3 SPLs formulation showed the highest EE percent among the formulations tested. The EE percent ranged from 55.8 ± 1.7% to 91.2 ± 2.1%. The addition of oleic acid to the F3 formulation also resulted in a slight increase in the EE percent. SPLs (F3 OA 1) containing oleic acid as the penetration enhancer (1%) gave the highest EE percent value of 91.2 ± 2.1%, which was statistically significant compared to the other formulations. The EE percent showed the following order: F3 OA 1 > F3 OA 0.5 > F3 > F3 PG 0.5 > F3 PG 1 > F4 > F2 > F5 > F1. The results also showed that the EE was higher in OA than in PG, which might be due to lipophilicity. OA is a lipophilic penetration enhancer, which increases the lipophilicity of vesicles and increases their ability to entrap lipophilic drugs like TCR. On the other hand, PG decreases the lipophilicity of vesicles, which results in a reduced encapsulation of the lipophilic substance.

### 3.2. Transmission Electron Microscopy (TEM)

**Figure 1:**
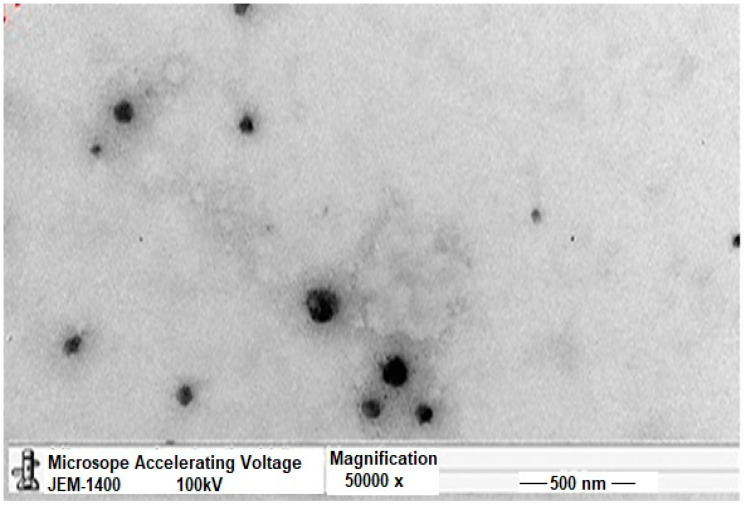
TEM image of selected TCR-loaded SPL formulation (F3 OA 1).

The TEM micrograph of the selected SPL formulation (F3 OA 1) is shown in Figure 1. The figure shows that the particles’ size of the SPL formulation was in the range of 200–300 nm. Dark and almost spherical vesicles were observed. This dark color may be due to the interaction with phosphotungstic acid, which was used to stain the vesicles [31].

### 3.3. Differential Scanning Calorimetry (DSC)

**Figure 2:**
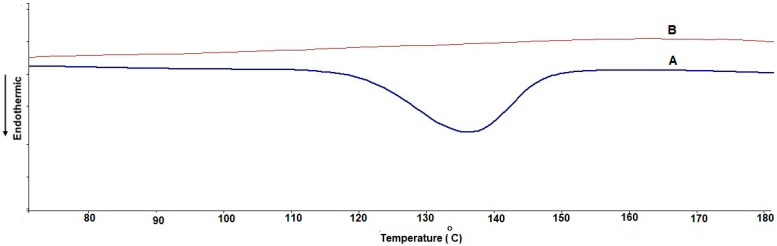
(A) Pure TCR drug used and (B) DSC thermograms of selected lyophilized TCR-loaded SPLs (F3 OA 1).

Figure 2 shows the thermograms of pure TCR and the selected lyophilized TCR-loaded SPL (F3 OA 1) formulation. The DSC thermogram of pure TCR shows a melting endotherm at 136.37 °C, which corresponds to its melting point. The endothermic peak of TCR is completely absent in the selected freeze-dried formulation, indicating that the drug is entrapped in the nanovesicular SPLs in amorphous form [32].

### 3.4. In Vitro Release of Different Permeation Enhancers Containing TCR–SPLs

**Figure 3:**
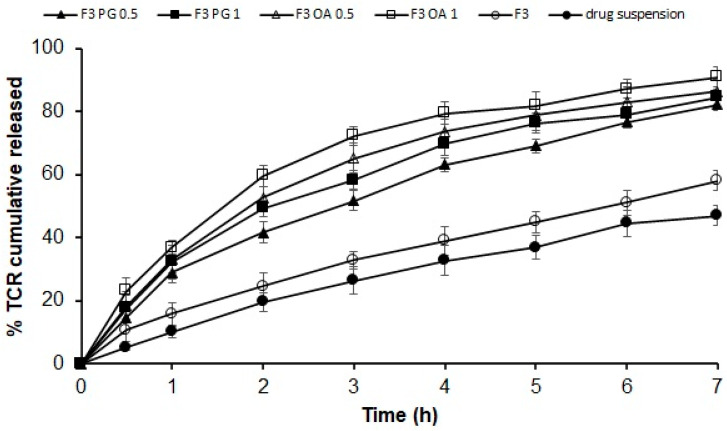
In vitro release data of different permeation enhancers containing TCR-loaded SPLs. Results (*n* = 3) are presented as mean ± SD.

The influences of different concentrations of propylene glycol and oleic acid on the in vitro release profiles of TCR from the SPL formulation (F3) are shown in Figure 3 and Table 3. SPL formulation F3 was selected for the study because it exhibited promising properties in terms of high entrapment efficiency (85.7 ± 1.4%), small vesicle size (240.7 ± 6.5 nm), and high zeta potential value (−22.2 ± 2.7 mV). TCR showed a slow release rate from drug suspension, with only 5.27 ± 0.95% released after 0.5 h and 47.03 ± 2.99% of the loaded drug released within the release period (7 h). In contrast, the release rate of the drug from the SPL formulation F3, from which 58.17 ± 2.99% was released after 7 h, was slightly increased. However, the addition of propylene glycol and oleic acid at concentrations of 0.5% and 1%, respectively, resulted in a significant improvement in the release of the drug from the SPL formulations (*p* < 0.05). The observed drug release rates from the F3 SPLs formulations containing 0.5% and 1% propylene glycol were 82.27 ± 2.04% and 84.57 ± 2.46%, respectively, while the release rates of 86.27 ± 4.07% and 90.97 ± 3.09% were observed for these formulations containing 0.5% and 1% oleic acid, respectively. The significantly increased in vitro release rates of TCR from F3 SPLs formulations containing 0.5% and 1% oleic acid compared to the formulations containing the same concentrations of propylene glycol (*p* < 0.05) might be due to the smaller vesicle size of the F3 SPLs formulations containing 0.5% and 1% oleic acid (276.1 ± 5.6 nm and 297.03 ± 7.6 nm, respectively) compared to the formulations containing the hydrophilic enhancer propylene glycol. In addition, the solubilizing effects of oleic acid and propylene glycol on the lipophilic drug, TCR, might act as a contributory factor on enhancing the drug’s in vitro release.

Table 3 illustrates the kinetic analysis of in vitro release data of TCR from the tested SPL formulations. The preference of the release mechanism is based on the correlation coefficient value. The release of TCR from these SPL formulations followed first-order kinetics. This indicates the dependence of the drug release on drug concentration, where the rate was concentration dependent, meaning the greater the concentration, the faster the process [33].

### 3.5. Ex Vivo Permeation of TCR-Loaded SPL Formula through Hairless Rat Skin

**Figure 4:**
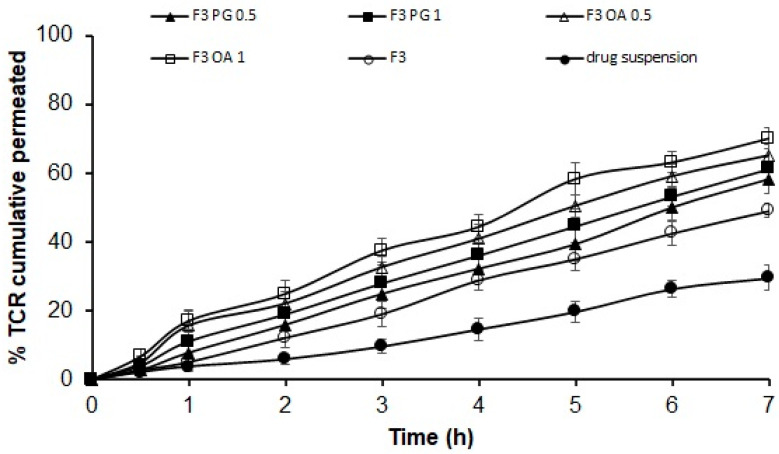
Ex vivo permeation data of different permeation enhancers containing TCR-loaded SPLs. Results (*n* = 3) are presented as mean ± SD.

Figure 4 and Table 3 show the ex vivo permeation data for TCR from different SPL formulations containing permeation enhancers. TCR showed a slow ex vivo permeation rate from drug suspension, with only 29.5 ± 3.67% of the loaded drug permeating in 7 h. In contrast, the drug showed a significant improvement in its permeation rate from the SPL formula F3, of which approximately 49.1 ± 2.08% permeated at 7 h. Oleic acid and propylene glycol at concentrations of 0.5% and 1% significantly (*p* < 0.05) improved the ex vivo permeation of TCR from formulation F3. The ex vivo permeation rates of the drug from the F3 SPLs formula containing 0.5% and 1% propylene glycol were 58.23 ± 3.4% and 61.37 ± 3.29%, respectively, whereas 65.17 ± 3.36% and 70.07 ± 3.06% were permeated by the hairless rat skin from this formulation containing 0.5% and 1% oleic acid, respectively. This may be due to the smaller vesicle size of the F3 SPLs formulation with 0.5% and 1% oleic acid (as mentioned previously in the in vitro release section) compared to the formulations with the hydrophilic enhancer propylene glycol. In addition, the incorporation of the highly lipophilic drug TCR into the hydrophobic core of the SPL formula with oleic acid may result in the improved permeation of the drug [34,35]. As a penetration enhancer, oleic acid (OA) increases skin permeability by selectively acting on extracellular lipids, which are the main control pathway for small molecule diffusion [36]. The drug also showed the enhancement of ex vivo permeation from the F3 formula via propylene glycol (PG). PG is a small polyhydric solvent molecule that penetrates the skin by itself, and its presence in the skin can alter the distribution of the drug between the SPL formula and the skin. If partitioning is increased, there will be an enhanced flux [37]. The incorporation of OA at concentrations of 0.5% and 1% increased the ex vivo permeation rate of the drug compared to the same concentrations of PG. Most findings on the use of oleic acid as a penetration enhancer showed that its skin penetration enhancing effect was associated with its mechanism to liquefy the stratum corneum lipids [31].

### 3.6. Characterization of TCR-Loaded SPL Gel Formulation

The appearance of TCR-loaded SPL gel formulation was smooth without any phase separation. It was homogeneous and clear without any particulate matter. The pH was 6.54 ± 0.124 which is in agreement with the pH of the skin, so no irritation is expected [38]. The spreadability value was 3.94 ± 0.153, indicating good spreadability with low shear [39].

### 3.7. In Vitro Release of TCR from SPL-Loaded Gel Formulation

**Figure 5:**
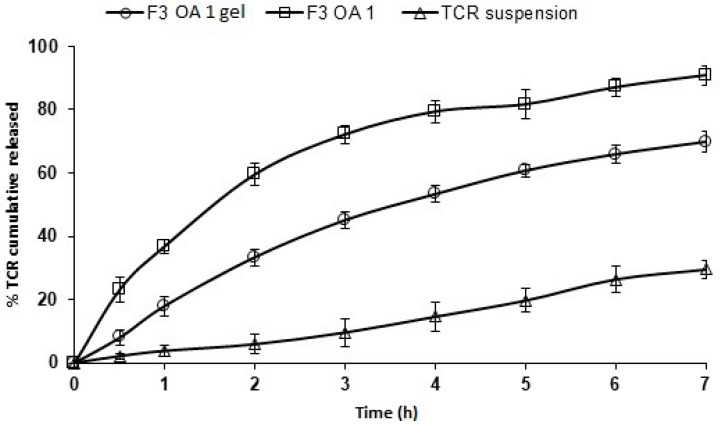
In vitro release of TCR from F3 OA 1 SPL gel, and F3 OA 1 SPLs (*n* = 3, mean ± SD).

Based on specific desirability in terms of a small vesicle size, a PDI of less than 0.3, the highest zeta potential value, the highest in vitro release, and ex vivo permeation, the TCR–SPLs formulation (F3 OA 1) containing oleic acid 1% was selected as the best formulation to be incorporated into a gel base for easy topical application. Figure 5 illustrates the in vitro release of TCR from the F3 OA 1 formulation, from the F3 OA 1 gel formulation, and from suspension. The TCR release rate from the F3 OA 1 gel formulation showed a slower release rate than the F3 OA 1 formulation. The gel formulation had a release rate of 69.87 ± 3.40% at 7 h with an initial release of 8 ± 2.5% at 0.5 h. This slow release may be due to release from nanovesicles and diffusion of the drug through the network structures of the gel, resulting in a controlled release model for the SPLs gel formulation loaded with TCR. On the other hand, the release of TCR from the suspension formulation was very low and not more than 30% released within 7 h. This slow release could be due to its poor water solubility.

### 3.8. Ex Vivo Permeation of TCR from SPLs-Loaded Gel Formulations

**Figure 6:**
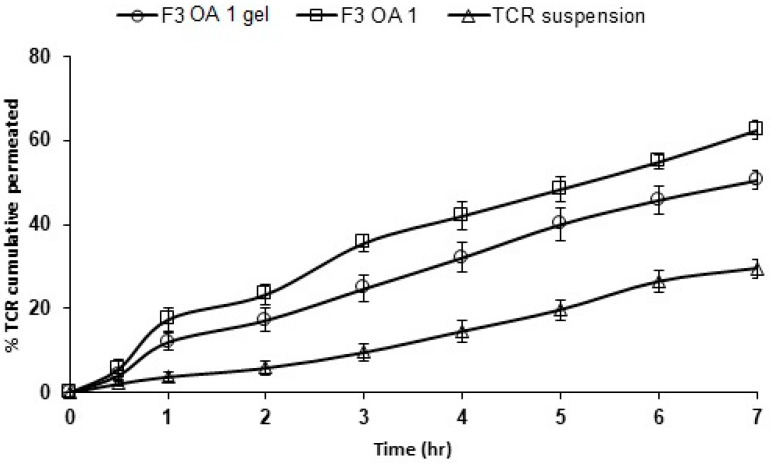
Ex vivo permeation of TCR from F3 OA 1 SPLs gel and F3 OA 1 SPLs (*n* = 3, mean ± SD) in rat skin.

The ex vivo permeation profiles of TCR from the F3 OA 1 formulation, the F3 OA 1 gel, and the suspension formulation are shown in Figure 6. It is evident that there is a favorable relationship between the in vitro release and ex vivo skin permeation of TCR from the topical formulations studied. A high permeation rate of the drug through the skin was observed for the F3 OA 1 formulation and the F 3 OA 1 gel formulation. The permeation parameters of TCR from F3 OA 1 SPLs and F3 OA 1 SPLs in HPMC gel through rat skin are shown in Table 4. The values of drug flux (Jss), permeation coefficient (P), and partition coefficient (Kp) were significantly higher in F3 OA 1 SPLs than in the gel base loaded with F3 OA 1 SPLs. The flux and permeation coefficient values were 36.79 ± 2.32 and 50.27 ± 3.06 (µg/cm2 h^−1^) and 7.36 ± 2.32 and 10.15 ± 3.06 (cm/h) for the F3 OA 1 SPLs gel and F3 OA 1 SPLs, respectively. The same conclusion was drawn for the distribution coefficient values, with the highest partition coefficient value (283 ± 3.06) obtained with F3 OA 1 SPLs. The figure also illustrates the permeation profile of TCR from its suspension form. The permeation was very low, indicating the poor permeability of the drug through skin, which is contributed to its large molecular weight.

### 3.9. Pharmacokinetic Studies

**Figure 7:**
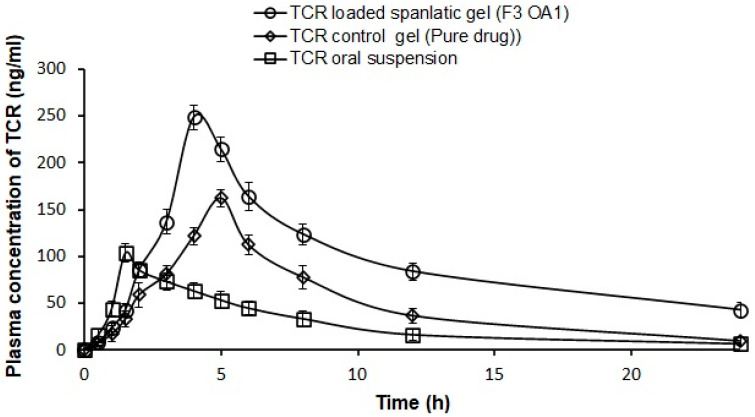
Plasma concentration–time profile of TCR from F3 OA 1 SPLs gel compared to TCR-loaded gel and oral drug suspension in rats. Results (*n* = 5) are presented as mean ± SD.

After the transdermal application of the TCR-loaded F3 OA 1 gel formulation, TCR-loaded gel, and oral TCR suspension to the rats, the mean plasma concentrations were calculated as a function of time, as shown in Figure 7. Plasma concentrations were found to increase with increasing time, reaching a maximum peak value at 4.00, 5.33, and 1.50 h for the TCR-loaded SPLs gel, TCR-loaded gel, and TCR suspension, respectively. The pharmacokinetic parameters are listed in Table 5. The Cmax and Tmax after the oral administration of the TCR suspension were 103.56 ± 9.55 ng/mL and 1.50 ± 0.00 h, respectively. In the case of the TCR-loaded SPLs gel, the Cmax (248.56 ± 12.95 ng/mL) and Tmax (4.00 ± 0.00 h) values were significantly (*p* < 0.05) different from both the oral administration of the TCR suspension and the TCR-loaded gel (Cmax; 163.20 ± 6.72 ng/mL and Tmax; 5.33 ± 0.58 h). The TCR-loaded SPLs gels exhibited approximately 2.4-fold Cmax and 1.52-fold Tmax compared with the oral TCR suspension. The biological half-life (t1/2) of TCR was prolonged to approximately 9.624 ± 0.93 h, compared with 5.70 ± 0.50 h and 4.80 ± 0.68 h for the TCR suspension and TCR-loaded gels, respectively. This behavior is supported by the high t1/2 values and significantly increased mean residence time (MRT) values obtained with the TCR-loaded SPLs gel (15.39 ± 0.79 h). The pharmacokinetic parameters found with the TCR-loaded SPLs gel were significantly different (*p* < 0.05) from those obtained with oral administration and the TCR-loaded gel. This may be due to the rapid absorption of drugs via the oral route, whereas drugs are absorbed slowly and continuously via the transdermal route [40]. Moreover, the AUC 0–24 (ng·h/mL) was significantly (*p* < 0.05) higher (3.36 and 1.81-fold) after the transdermal application of the TCR-loaded SPLs gel compared to oral administration and the TCR-loaded gel, respectively. This effect was attributed to the fact that the TCR-loaded SPLs gel maintained the TCR concentration in the range of pharmacological effect for a longer period of time. The observed high AUC values of the TCR-loaded SPLs gel delivery system indicate the increased bioavailability of TCR in rats compared with oral administration and the TCR-loaded gel.

## 4. Conclusions

The use of an adapted ethanol injection method was beneficial for the preparation of TCR–SPL nanovesicles containing propylene glycol and oleic acid as penetration enhancers. Moreover, a correlation between the in vitro release and ex vivo permeation of TCR was obtained. The results show that OA performed better than PG in improving the permeation of the lipophilic drug TCR, which is due to its lipophilic nature that can affect the structure of the stratum corneum and improve the permeation of the drug. In addition, the plasma concentrations and pharmacokinetic parameters of the SPLs-loaded gel base (F3 OA 1) were significantly increased compared to the TCR-loaded gel and oral drug suspension, which may lead to an increase in transdermal bioavailability.

## Figures and Tables

**Table 1:** Composition of different TCR-loaded SPL formulations.

Formula No.	Span 60 (mg)	Tween 80 (mg)	Tacrolimus (mg)	Permeation Enhancer (%)	
				Propylene Glycol (PG)	Oleic Acid (OA)
F1	85	15	5	-	-
F2	80	20	5	-	-
F3	75	25	5	-	-
F4	70	30	5	-	-
F5	50	50	5	-	-
F3 PG 0.5	75	25	5	0.5	-
F3 PG 1	75	25	5	1	-
F3 OA 0.5	75	25	5	-	0.5
F3 OA 1	75	25	5	-	1

**Table 2:** Characterization of the formulated TCR-loaded SPLs.

Formulation Code	EE% (Mean ± SD)	Vesicle Size (nm ± SD)	PDI (±SD)	Zeta Potential (mV ± SD)
F1	55.8 ± 1.7	38.1 ± 7.01	0.112 ± 0.0	−20.16 ± 2.8
F2	71.1 ± 1.5	143.8 ± 6.3	0.176 ± 0.0	−16 ± 1.30
F3	85.7 ± 1.4	240.7 ± 6.5	0.213 ± 0.1	−22.2 ± 2.7
F4	77.8 ± 2.9	360.8 ± 6.6	0.311 ± 0.1	−19.3 ± 2.05
F5	65.1 ± 2.1	407.3 ± 8.4	0.386 ± 0.1	−18.9 ± 1.8
F3 PG 0.5	84.7 ± 2.1	301.6 ± 10.1	0.281 ± 0.1	−17.4 ± 1.9
F3 PG 1	82.7 ± 2.4	375.5 ± 6.1	0.327 ± 0.1	−20.4 ± 1.4
F3 OA 0.5	89.5 ± 1.9	276.1 ± 5.6	0.265 ± 0.0	−18.4 ± 1.05
F3 OA 1	91.2 ± 2.1	297.03 ± 7.6	0.281 ± 0.1	−24.5 ± 0.7

**Table 3:** In vitro release, the kinetic parameters and ex vivo permeation of different permeation enhancers containing TCR–SPLs (*n* = 3, mean ± SD).

Formulation Code	% In Vitro Cumulative Released after 7 h	Linear Regression Analysis Using Correlation Coefficient R^2^ According to				% Ex Vivo Cumulative Permeated after 7 h
		Zero	First	Second	Higuchi	
F3	58.17 ± 2.99	0.975	0.994	0.979	0.984	49.10 ± 2.08
F3 PG 0.5	82.27 ± 2.04	0.940	0.996	0.936	0.993	58.27 ± 3.40
F3 PG 1	84.57 ± 2.46	0.931	0.993	0.975	0.992	61.37 ± 3.29
F3 OA 0.5	86.27 ± 4.07	0.877	0.987	0.979	0.982	65.17 ± 3.36
F3 OA 1	90.97 ± 3.09	0.846	0.984	0.933	0.974	70.07 ± 3.06
Drug suspension	47.03 ± 2.99	0.979	0.994	0.993	0.970	29.50 ± 3.67

**Table 4:** Ex vivo permeation parameters of TCR from selected SPLs (F3 OA 1) and F3 OA 1 gel formula (*n* = 3, mean ± SD).

Formulation Code	Flux (J_ss_) (µg/cm^2^ h^−1^) × 10^4^	Permeability Coefficient (P) (cm/h) × 10^−6^	Partition Coefficient (K_P_) ×10^4^
F3 OA 1	50.27 ± 3.06	10.15 ± 3.06	283 ± 3.06
F3 OA 1 gel	36.79 ± 2.32	7.36 ± 2.32	155 ± 2.32

**Table 5:** Pharmacokinetic parameters of TCR after administration of TCR-loaded SPLs gel formula (F3 OA 1 gel), TCR-loaded gel, and TCR oral suspension in rat plasma.

Pharmacokinetic Parameters	Oral TCR Suspension	TCR-Loaded Gel (Pure Drug)	TCR-Loaded SPLs Gel (F3 OA 1 Gel)
t_½_ (h)	5.70 ± 0.50	4.80 ± 0.68	9.62 ± 0.93 *
T_max_ (h)	1.50 ± 0.00	5.33 ± 0.58	4.00 ± 0.00 *
C_max_ (ng/mL)	103.56 ± 9.55	163.20 ± 6.72	248.56 ± 12.95 *
AUC_0–7_ (ng·h/mL)	668.78 ± 26.26	1243.60 ± 121.56	2246.01 ± 125.06 *
AUC_0–∞_ (ng·h/mL)	720.43 ± 35.28	1310.45 ± 154.02	2838.60 ± 227.00 *
MRT_0–∞_ (h)	8.70 ± 0.73	9.01 ± 0.78	15.39 ± 0.79 *

* Statistically significant (*p* < 0.05).

## Data Availability

The data presented in this study are available on request from the corresponding author.
